# The Impact of Genomic Burden on Dementia Caregiving: A Scoping Review

**DOI:** 10.1093/geroni/igaf122.4026

**Published:** 2025-12-31

**Authors:** Wenxin Bian, Jing Wang, Jamie Conklin, Xiangyu Zhang, Chloe Mitchell, Aubrie Woodward, Rose Mary Xavier

**Affiliations:** University of North Carolina at Chapel Hill, Chapel Hill, North Carolina, United States; University of New Hampshire, Durham, New Hampshire, United States; University of North Carolina at Chapel Hill, Chapel Hill, North Carolina, United States; University of New Hampshire, Durham, New Hampshire, United States; University of New Hampshire, Durham, New Hampshire, United States; University of New Hampshire, Durham, New Hampshire, United States; UTHealth Houston, Houston, Texas, United States

## Abstract

**Background:**

Although genomic technologies are increasingly used in dementia risk assessment, diagnosis and treatment, the impact of genomic burden in persons living with dementia (PWLD) on caregiving remains largely unexamined.

**Purpose:**

We investigated the impact of genomic burden on dementia caregiving and identified gaps to inform future practice and policy on dementia care.

**Methods:**

We searched PubMed, APA PsycInfo, and CINAHL through April 2025 for peer-reviewed studies examining caregiver outcomes related to genomic burden in PLWD. Two reviewers independently screened articles, resolving discrepancies by consensus. Data were extracted and thematically analyzed.

**Results:**

Of 708 studies identified, 82 underwent full-text screening. 5 studies that met inclusion criteria were conducted in the UK (n = 1) and Brazil (n = 4). Investigated genomic factors included APOE (n = 4), LDLD (n = 1), ACE (n = 1), and MAO-A (n = 1) variants. Care recipients, mainly with late-onset Alzheimer’s disease, represented a range of dementia severity from probable to severe. Caregivers were primarily family members. Caregiving outcomes included caregiving burden and caregiving distress.

**Conclusion:**

Available evidence to determine the impact of genomic burden in PWLD on caregiving outcomes is limited, but they point to the mediating role of neuropsychiatric symptoms. Longitudinal studies are needed to assess how genomic burden influences caregiving burden. Intervention studies integrating genomic information and/or genetic counseling in dementia care models could clarify clinical utility as well as the psychosocial effects of genomic risk disclosure on the caregiving process.

